# Supplementary material for: CD26-negative and CD26-positive tissue-resident fibroblasts contribute to functionally distinct CAF subpopulations in breast cancer
Source: Nat Commun. 2023 Jan 12;14:183. doi: 10.1038/s41467-023-35793-w (PMC9837080; doi:10.1038/s41467-023-35793-w)
Supplement: Supplementary file 1 — Supplementary Information [file 41467_2023_35793_MOESM1_ESM.pdf]

Supplemental figure 1

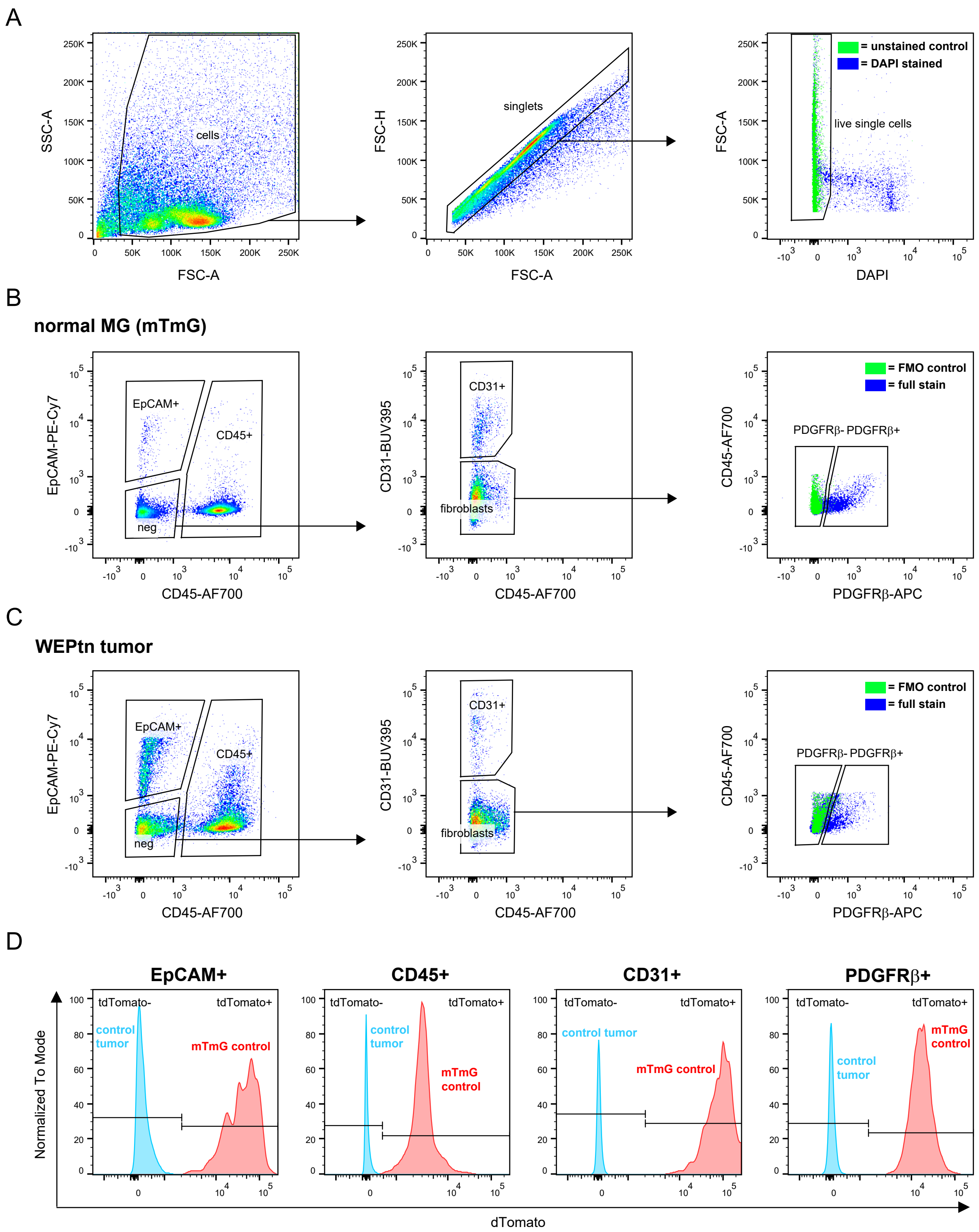

Supplemental figure 1: Gating strategy for WEPtn and WEH1047R tumors and controls from transplantation studies. A) General gating done on all samples for cells, singlets and live single cells. B) Normal mammary glands derived from mTmG mice were stained with EpCAM-PE-Cy7, CD45-AF700, CD31-BUV395 and PDGFRβ-APC. Mammary epithelium was defined as EpCAM+/CD45-/CD31-/PDGFRβ-. Immune cells were defined as CD45+/CD31-/EpCAM-. Endothelial cells were defined as CD31+/EpCAM-/CD45-. Fibroblasts were defined as EpCAM-/CD45-/CD31-/PDGFRβ+. C) WEPtn and WEH1047R derived ILC tumors (WEPtn shown here) were stained with EpCAM-PE-Cy7, CD45-AF700, CD31-BUV395 and PDGFRβ-APC and used the same markers to identify epithelium, immune cells, endothelium and fibroblasts as in panel B. In panel B and C fluorescence-minus-one (FMO) control stained samples (all antibodies except PDGFRβ) are shown in green and full stained samples are shown in blue. D) tdTomato expression in indicated cell populations from mTmG control mice and WEPtn mice with gates that define tdTomato+ (in red) and tdTomato- (in blue) cells for each cell population.

Supplemental figure 2

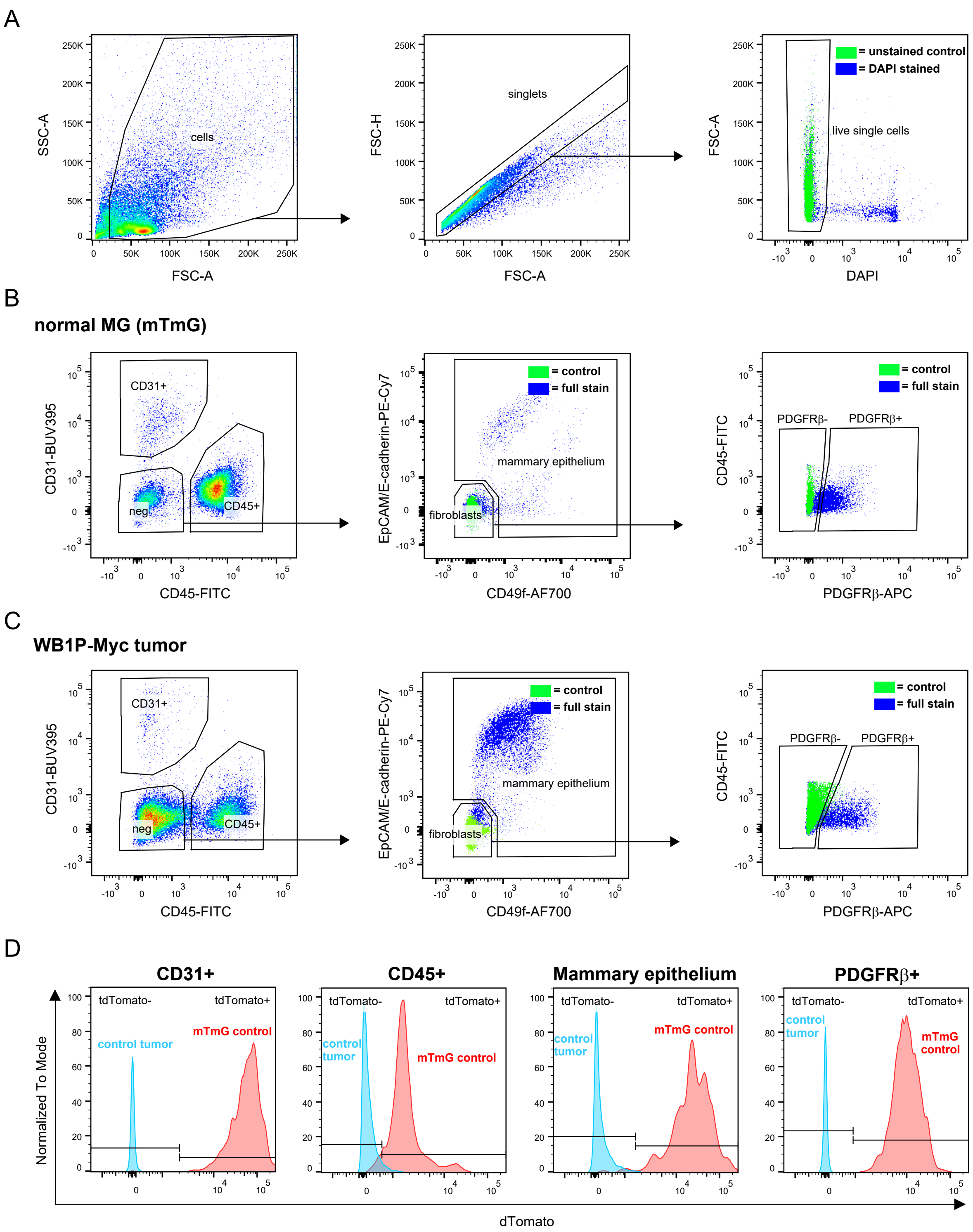

Supplemental figure 2: Gating strategy for WB1P and WB1P-Myc tumors and controls from transplantation studies. A) General gating done on all samples for cells, singlets and live single cells. B) Normal mammary glands derived from mTmG mice were stained with CD31-BUV395, CD45-FITC, EpCAM-PE-Cy7, E-cadherin-PE-Cy7, CD49f-AF700 and PDGFRβ-APC. Mammary epithelium was defined as EpCAM/E-Cadherin+/CD49f+/low/CD45-/CD31-/PDGFRβ-. Immune cells were defined as CD45+/CD31-/EpCAM/E-Cadherin-. Endothelial cells were defined as CD31+/EpCAM/E-Cadherin-/CD45-. Fibroblasts were defined as EpCAM/E-Cadherin-/CD49f-/CD45-/CD31-/PDGFRβ+. C) WB1P and WB1P-Myc derived TNBC tumors (WB1P-Myc shown here) were stained with CD31-BUV395, CD45-FITC, EpCAM-PE-Cy7, E-cadherin-PE-Cy7, CD49f-AF700 and PDGFRβ-APC and used the same markers to identify epithelium, immune cells, endothelium and fibroblasts as in panel B. In panel B and C control stained samples (only CD31-BUV395 and CD45-FITC) are shown in green and full stained samples are shown in blue. D) tdTomato expression in indicated cell populations from mTmG control mice and WB1P-Myc mice with gates that define tdTomato+ (in red) and tdTomato- (in blue) cells for each cell population.

Supplemental figure 3

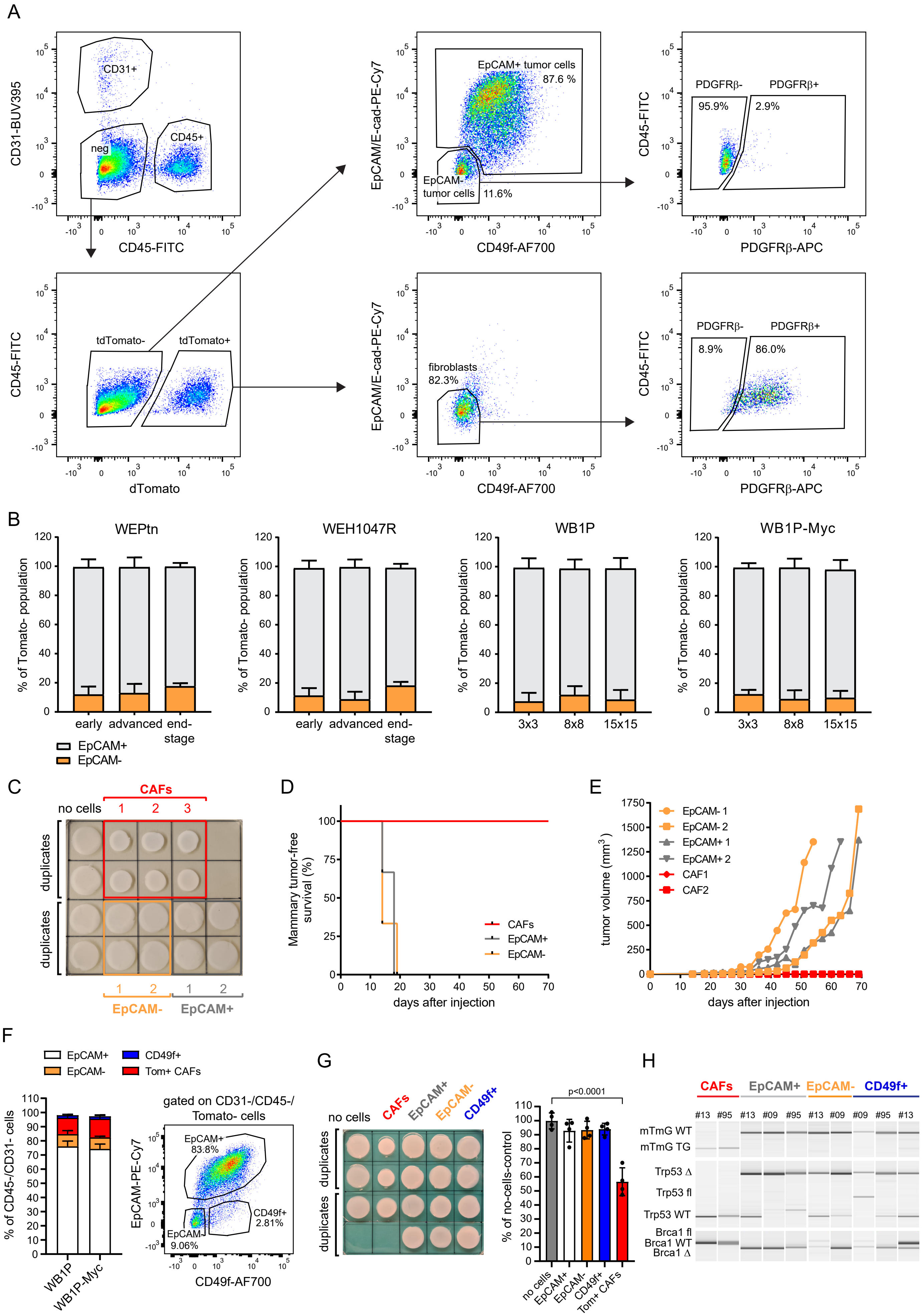

Supplemental figure 3: EMT tumor cells are present in breast cancer mouse models but lack CAF marker expression and function. A) Gating strategy used to identify EpCAM-positive tumor cells (EpCAM+) and tumor cells that have lost EpCAM expression (EpCAM-). After exclusion of endothelial cells (CD31+) and immune cells (CD45+) remaining cells were divided into tdTomato-negative cells (tdTomato-, coming from transplanted tumor tissue) and tdTomato-positive cells (tdTomato+, coming from recipient mTmG host). EpCAM-positive tumor cells were defined as tdTomato-/EpCAM+/E-Cadherin+/CD49f+/low. EpCAM-negative tumor cells were defined as tdTomato-/EpCAM+/E-Cadherin-/CD49f-/PDGFRβ-. tdTomato+ cells consisted primarily of fibroblasts as they lacked expression of epithelial/basal markers (EpCAM, E-Cadherin, CD49f), but were positive for PDGFRβ. B) Quantification of EpCAM+ tumor cells and EpCAM- tumor cells in all MMEC transplanted mice (n=5 mice per time point). Data represented as mean ± SEM. C) Collagen contraction assay with EpCAM+ tumor cells (n=2), EpCAM- tumor cells (n=2) and tdTomato+ CAFs (n=3) isolated from WB1P-Myc MMEC transplanted mTmG mice. D) Mammary tumor-free survival of FVB/n mice orthotopically injected with EpCAM+ tumor cells, EpCAM- tumor cells or tdTomato+ CAFs (n=2 mice per group). E) Tumor volumetric measurements of FVB/n mice orthotopically injected with EpCAM+ tumor cells, EpCAM- tumor cells or tdTomato+ CAFs (n=2 mice per group). F) Representative FACS plot and quantification of the distribution of EpCAM+ tumor cells, EpCAM- tumor cells and EpCAM-/CD49f+ tumor cells, n=4 mice for WB1P-Myc and n=5 mice for WB1P. Data shown as mean ± SEM. G) Representative image and quantification of three-day collagen contraction assay with tdTomato+ CAFs, EpCAM+ tumor cells, EpCAM- tumor cells and EpCAM-/CD49f+ tumor cells. Results shown are from 4 independent experiments with similar outcome. H) PCR analysis of mTmG reporter transgene (TG) or mTmG wildtype (WT) alleles. Trp53 floxed, wildtype or deleted alleles and Brca1 floxed, wildtype or deleted alleles in CAFs (n=2), EpCAM+ tumor cells (n=3), EpCAM- tumor cells (n=2) and EpCAM-/CD49f+ tumor cells (n=3).

Supplemental figure 4

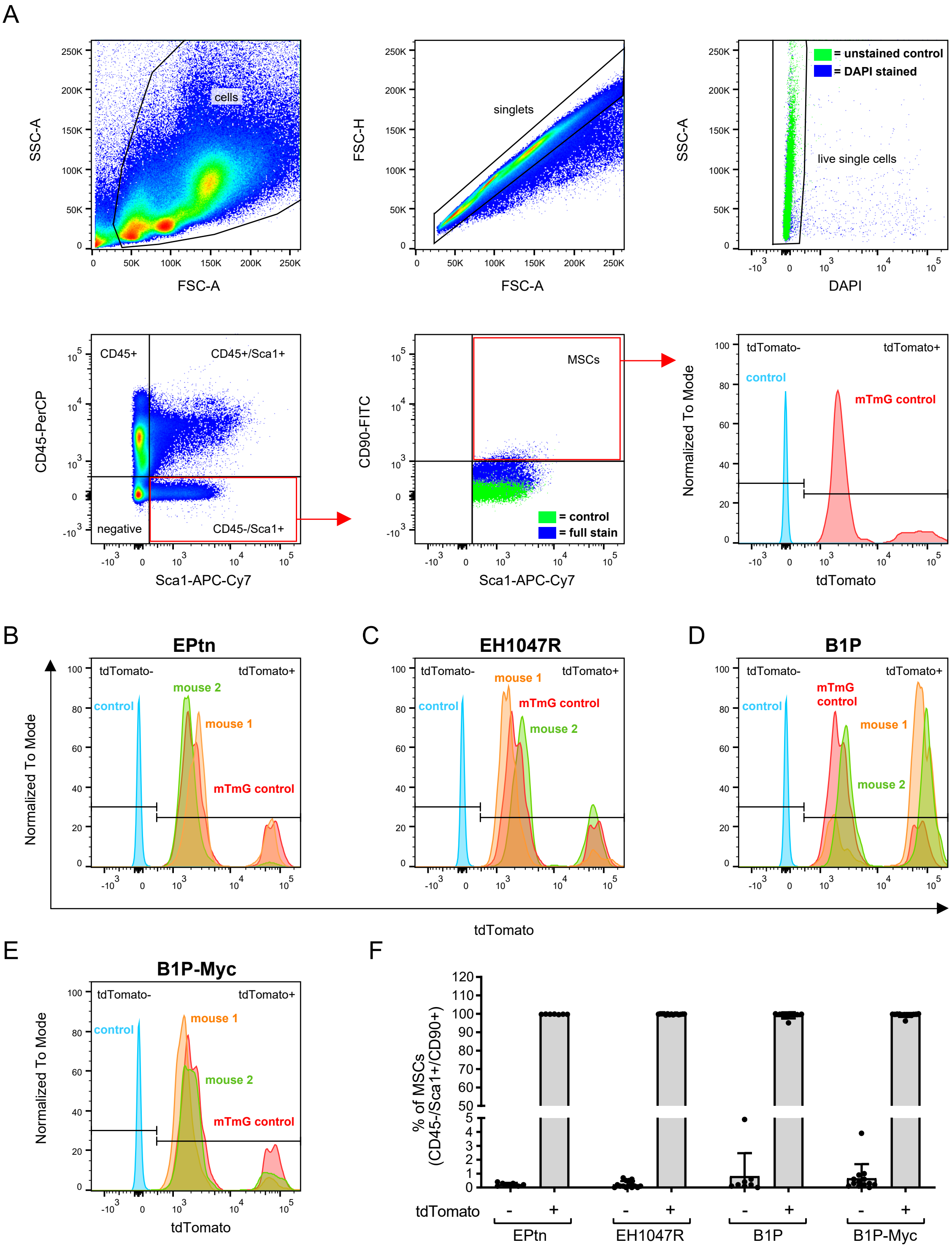

Supplemental figure 4: Efficient bone marrow transplantation shows tdTomato+ BM-MSCs. A) Gating strategy to define to and validate tdTomato expression in bone marrow mesenchymal stem cells (BM-MSCs). Bone marrow was harvested from FVB/n control mice, control mTmG mice and mTmG bone marrow-transplanted EPtn, EH1047R, B1P and B1P-Myc mice and stained with CD45-PerCP, Sca1-APC-Cy7 and CD90-FITC. MSCs were defined as CD45-/Sca1+/CD90 high. B-E) Representative flow cytometry plots showing tdTomato expression in mTmG control mice (in red), FVB/n control mice (in blue) and two mTmG bone marrow transplanted mice (in orange and green) of the indicated tumor mouse models. Panel B shows EPtn, panel C shows EH1047R, panel D shows B1P and panel E shows B1P-Myc. F) Quantification of tdTomato- and tdTomato+ BM-MSCs in bone marrow-transplanted mice of the indicated tumor models (EPtn n=7 mice, EH1047R n=15, B1P n=8 mice and B1P-Myc n=13 mice). Data represented as mean ± SEM.

Supplemental Figure 5

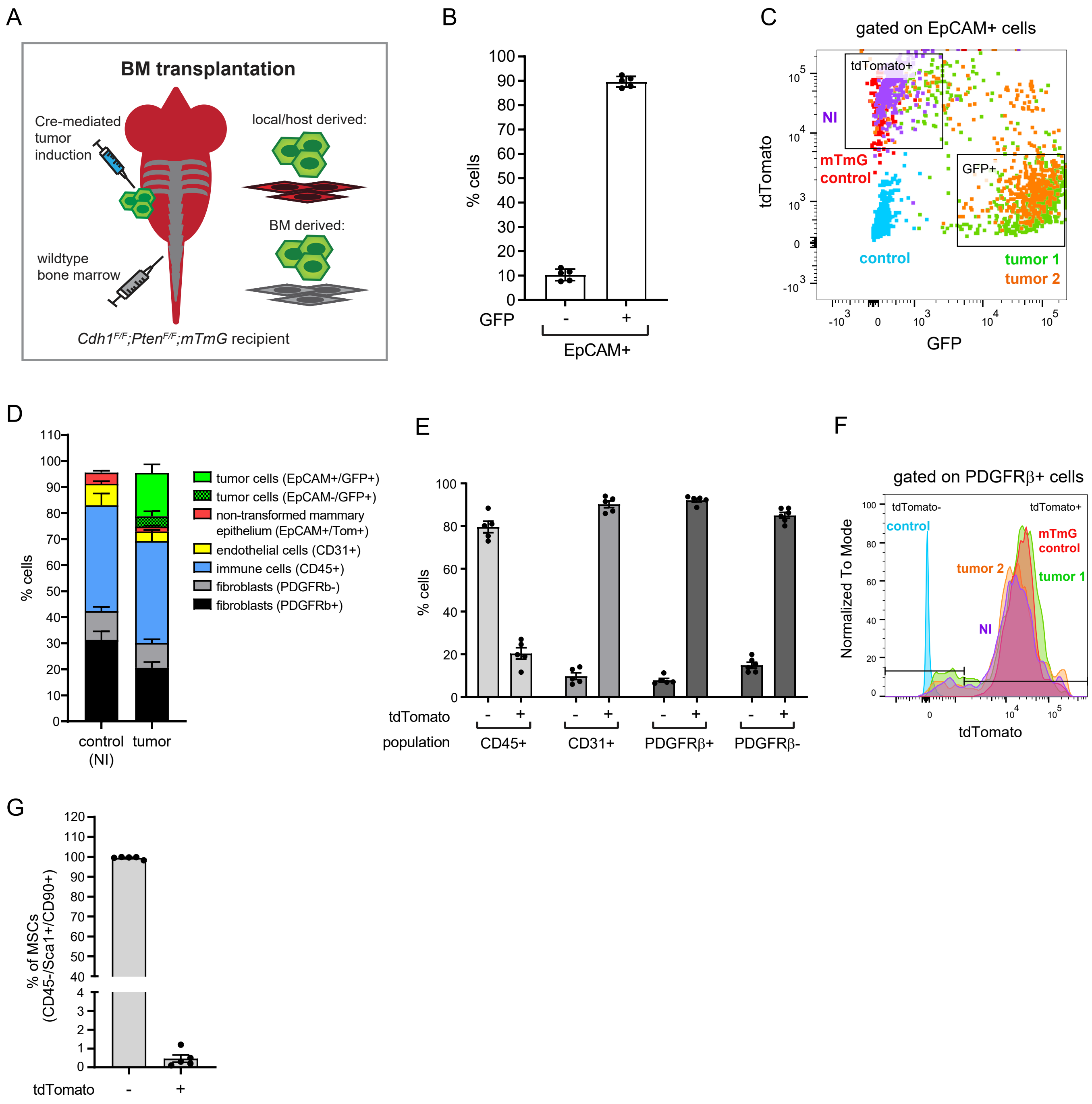

Supplemental figure 5: Bone marrow transplantations in EPtn;mTmG mice confirm findings that BM-MSCs do not contribute to the population of CAFs in ILC. A) Schematic representation of experimental set-up and its potential outcomes. B) Quantification of GFP expression within the mammary epithelium compartment (EpCAM+) in established tumors 18 weeks after lenti-cre injection (n=5 mice). Data represented as mean ± SEM. C) Representative flow cytometry plot of tdTomato and GFP expression of EpCAM+ mammary epithelial cells of FVB/n control mice (control, in blue), control non-intraductal injected EPtn;mTmG mice (NI, in purple) and two EPtn;mTmG mice after lenti-cre intraductal injection (18 weeks post injection, tumor 1 and 2 in green and orange). D) Distribution of cell populations in control non-intraductal injected EPtn;mTmG mice (NI, n=4 mice) and tumor-bearing mice (18 weeks post injection, n= 5 mice). Data represented as mean ± SEM. E) Quantification of tdTomato expression in indicated intratumoral cell populations harvested from EPtn;mTmG mice 18 weeks after intraductal lenti-cre injection (n=5 mice). Data represented as mean ± SEM. F) Representative flow cytometry plot of tdTomato expression of PDGFRβ+ fibroblasts of FVB/n control mice (control in blue), control non-intraductal injected EPtn;mTmG mice (NI, in purple) and two EPtn;mTmG mice after lenti-cre intraductal injection (18 weeks post injection, tumor 1 and 2 in green and orange). G) tdTomato expression in bone marrow of FVB/n bone marrow-transplanted EPtn;mTmG mice at time of tumor harvest (18 weeks after intraductal injection, n=5 mice). Data represented as mean ± SEM.

Supplemental figure 6

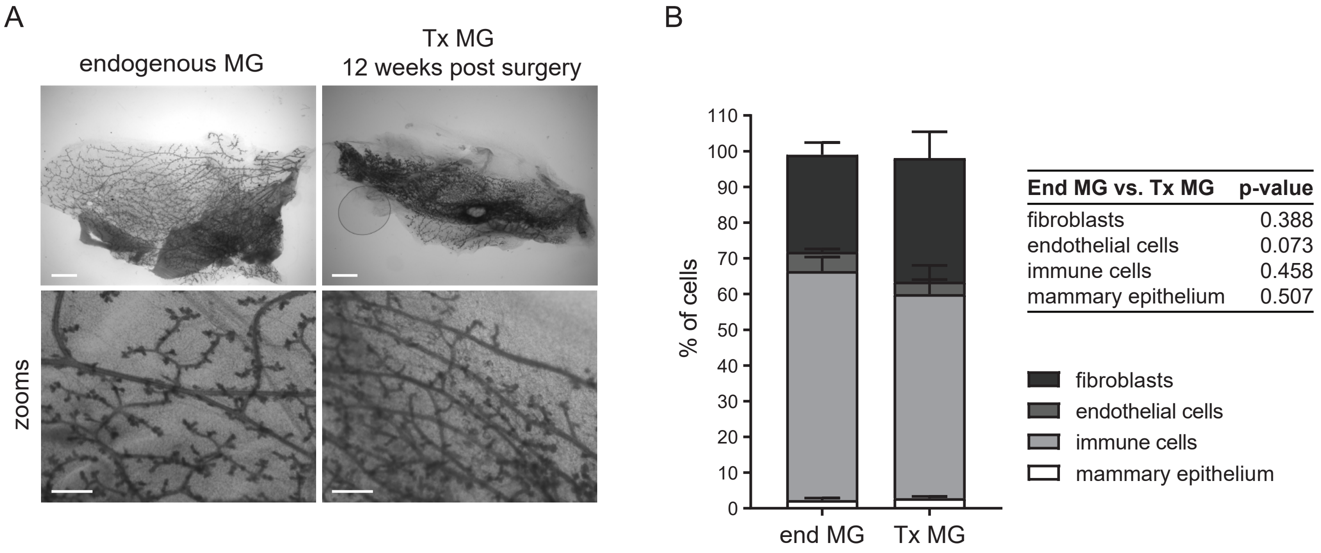

Supplemental figure 6: Morphology and cellular composition of whole mammary gland transplanted tissue resembles endogenous mammary glands. A) Representative images (n=3 biological replicates with similar outcome) of carmine stainings performed on endogenous and transplanted third whole mammary gland 12 weeks post-surgery. Scale bars in overview images are 1 mm. Scale bars in zoomed images are 250 um. B) Quantification of cellular composition of endogenous and transplanted mammary glands as determined by flow cytometry (n=5 mice per group). Mammary epithelial cells were defined as EpCAM+/CD31-/CD45-/PDGFRβ-. Endothelial cells were defined as CD31+/CD49f+/EpCAM-/CD45-. Immune cells were defined as CD45+/CD31-/EpCAM-. Fibroblasts were defined as EpCAM-/CD45-/CD31-/PDGFRβ+. Data represented as mean ± SEM. Statistical significance was determined using a two-tailed student's t-test.

Supplemental figure 7

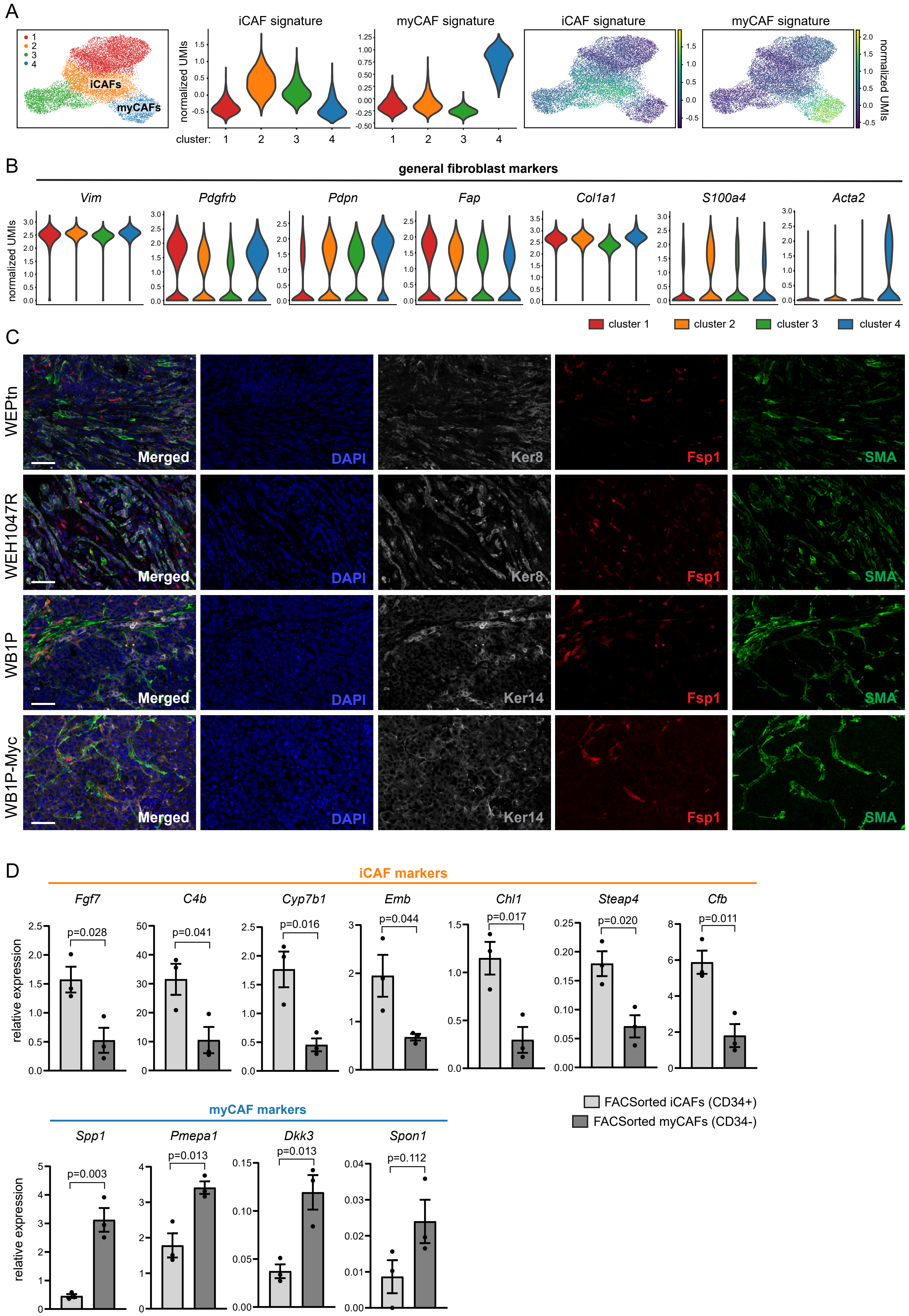

Supplemental figure 7: Gene expression signatures, FSP1 and SMA can distinguish between iCAFs and myCAFs. A) Clustering of all time points in one UMAP with violin and UMAP representations of iCAF and myCAF gene expression signatures. B) Violin representation of the expression of general fibroblast markers Vimentin (*Vim*), *Pdgrfb*, podoplanin (*Pdpn*), *Fap*, *Col1a1, S100a4* (Fsp1) and *Acta2* (SMA). C) Immunofluorescent stainings of end-stage tumors derived from WEPtn, WEH1047R, WB1P and WB1P-Myc for iCAFs (Fsp1), myCAFs (SMA) and tumor cells (Keratin 8 in WEPtn and WEH1047R and Keratin 14 in WB1P and WB1P-Myc). Nuclei are stained with Dapi. Scale bars are 50 um. Representative results are shown from two independent experiments with similar outcome. D) qPCR analysis of iCAF and myCAF markers in CD34+ and CD34- CAFs isolated from ILCs derived from WEPtn mice (n=3). Expression relative to *Gapdh* control. Data represented as mean ± SEM. Statistical significance was determined using two-tailed student's t-test.

Supplemental figure 8

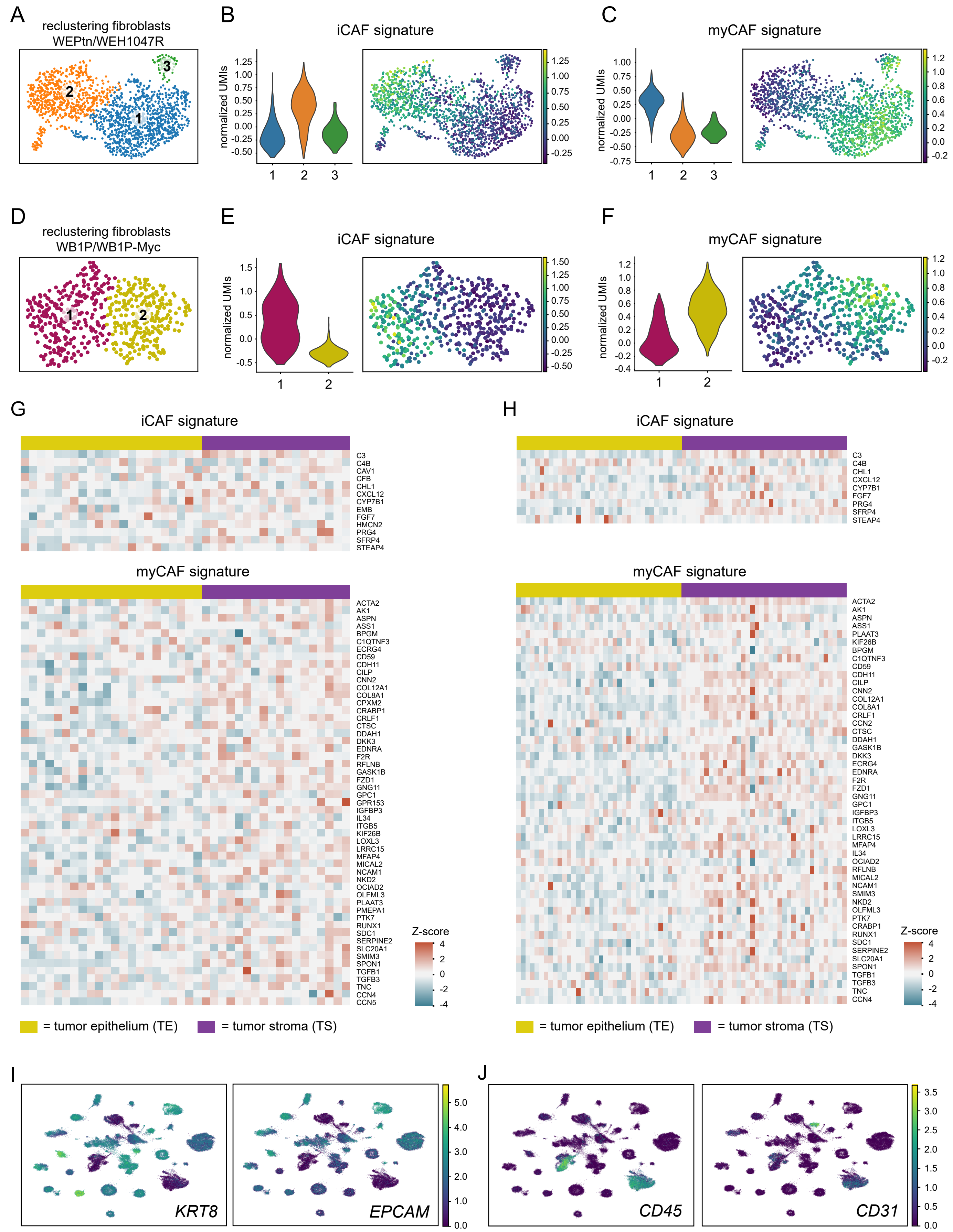

Supplemental figure 8: iCAFs and myCAFs are present in mouse models of TNBC and ILC. A) Reclustering of *Col1a1*-positive fibroblast cluster from WEPtn/WEH1047R dataset, with exclusion of *Rgs5*+ pericytes. B) iCAF gene expression signature in reclustered CAFs, data shown as violin plot and UMAP. C) myCAF gene expression signature in reclustered CAFs, data shown as violin plot and UMAP. D) Reclustering of *Col1a1*-positive fibroblast cluster from WB1P/WB1P-Myc dataset, with exclusion of RGS5+ pericytes. E) iCAF gene expression signature in reclustered CAFs, data shown as violin plot and UMAP. F) myCAF gene expression signature in reclustered, data shown as violin plot and UMAP. G) iCAF and myCAF gene expression signatures in laser-microdissected human ILC samples (n=17) separated into tumor epithelium (marked in yellow) and tumor stroma (marked in purple). H) iCAF and myCAF gene expression signatures in laser-microdissected human IDC samples (n=36) separated into tumor epithelium (yellow) and tumor stroma (purple). I) Expression of tumor epithelial markers (*EpCAM* and Keratin 8 (*KRT8*) in single-cell dataset of human breast cancers (n=34). J) Expression of *CD31* (endothelial cells) and *CD45* (immune cells).

Supplemental figure 9

A

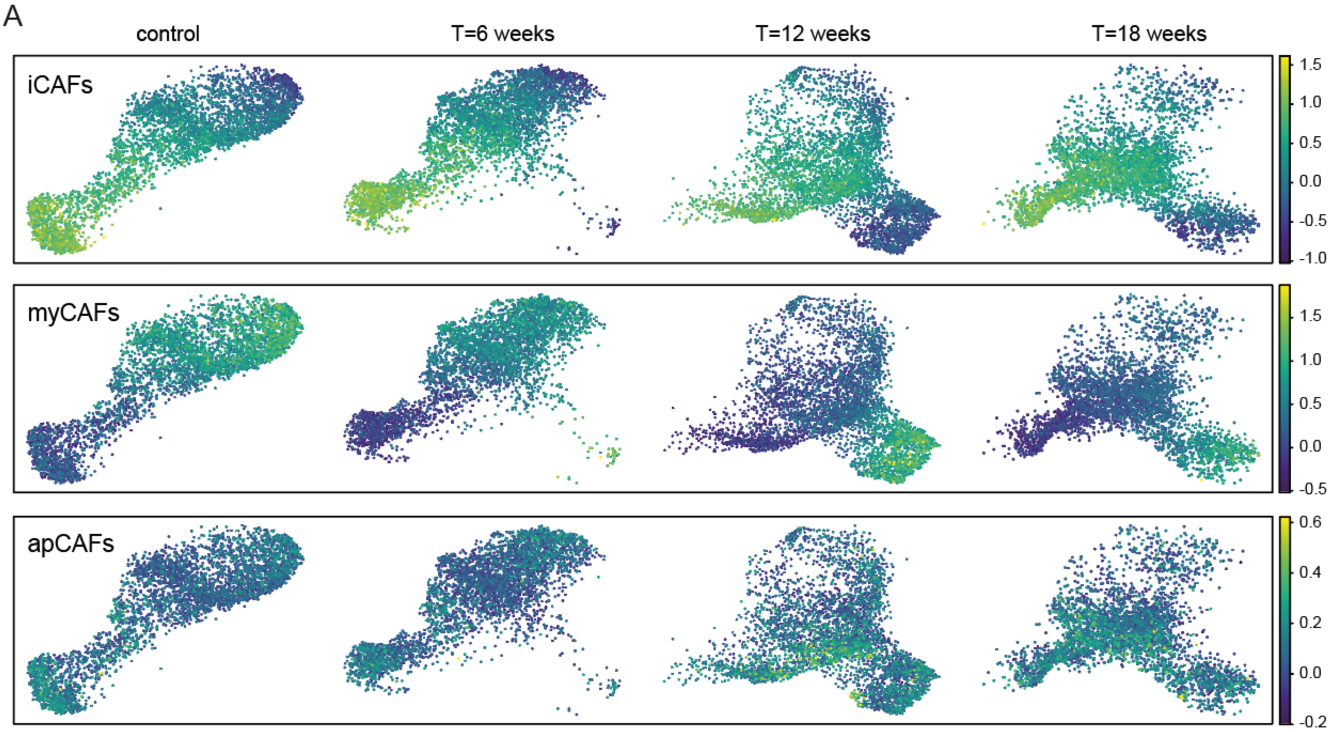

B

PDAC iCAF signature		
Dpt	*Scara3*	*Ly6a*
Ifi205	*Pcolce2*	*Ly6c1*
Gsn	*Clec3b*	*Adamts5*
Fifg	*Efemp1*	*Tnxb*
Svep1	*Ifi27l2a*	*C4b*
Plpp3	*Col14a1*	*C3*

PDAC myCAF signature		
Serpine2	*Crlf1*	*Sdc1*
Col15a1	*Thy1*	*Cthrc1*
Tnc	*Tagln*	*Col8a1*
Sparcl1	*Col12a1*	*Thbs2*
Spp1	*Col1a1*	*Acta2*
Tgfb1	*Cxcl14*	

PDAC apCAF signature		
Slpi	*Lgals7*	*Krt8*
Ptgis	*Spint2*	*Cxadr*
Nkain4	*Dmkn*	*Ezr*
Hspb1	*Saa3*	*H2-Ab1*
Cav1	*Clu*	*Cd74*
Arhgdib	*Slc9a3r1*	*Fth1*

C D

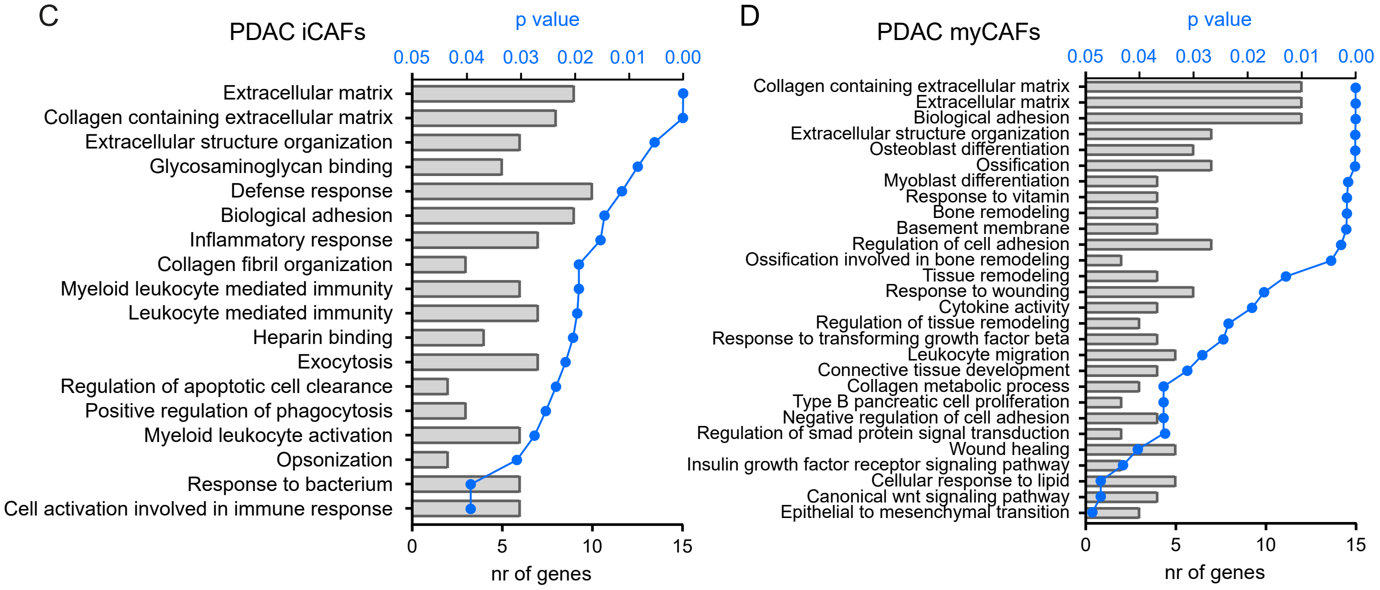

E F

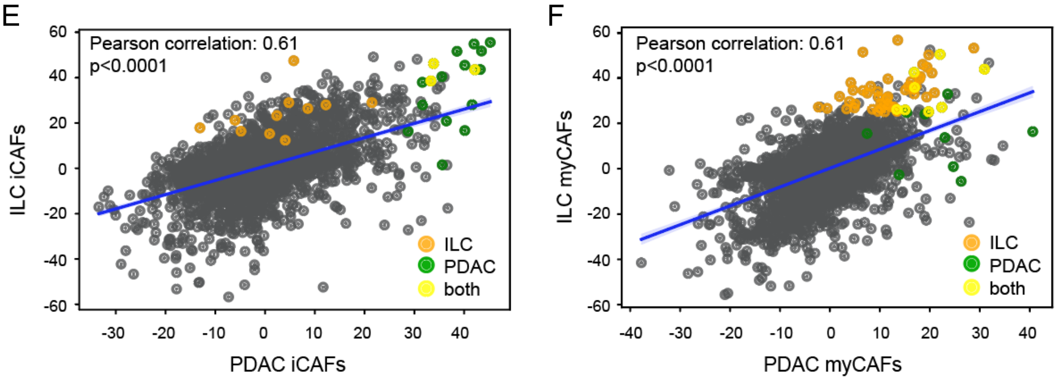

Supplemental figure 9: PDAC iCAFs and myCAFs show significant overlap in gene expression and function with ILC iCAFs and myCAFs. A) UMAP plots of PDAC iCAF, myCAF and apCAF gene signatures plotted on single cell dataset of NFs and CAFs isolated from EPtn mice. B) Gene signatures of PDAC iCAFs, myCAFs and apCAFs. C and D) Gene ontology analysis of the genes that define PDAC iCAFs (panel C) and myCAFs (panel D). Statistical significance was determined using Fisher's exact test with multiple comparison correction according to Benjamini-Hochberg. E) Pearson correlation between gene expression of PDAC iCAFs and ILC iCAFs beyond the gene signatures. F) Pearson correlation between gene expression of PDAC myCAFs and ILC myCAFs beyond the gene signatures. In panel E and F the gene-signature defining genes are highlighted in orange (ILC), green (PDAC) or yellow (both). All other genes expressed by iCAFs and myCAFs are shown in grey.

Supplemental figure 10

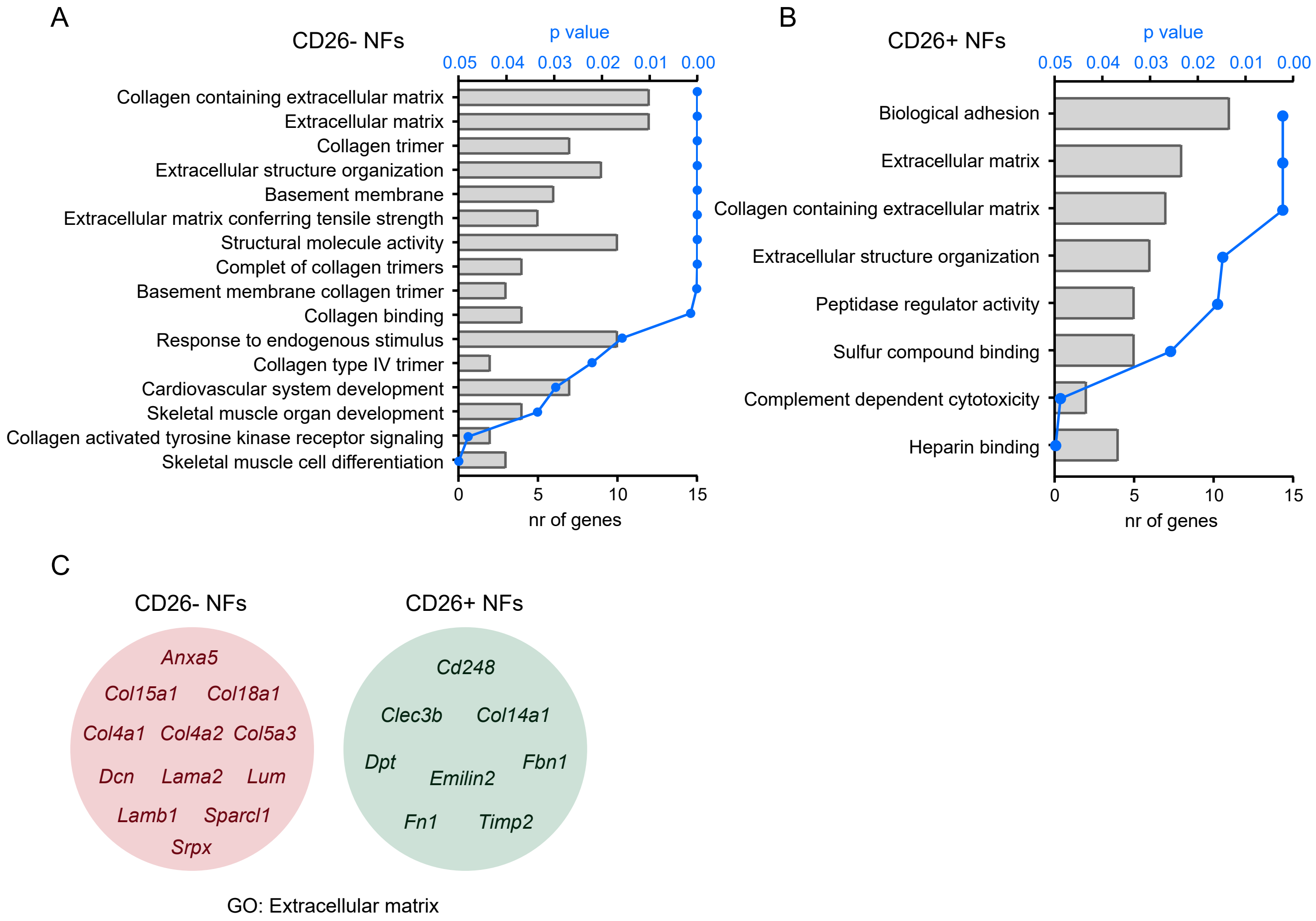

Supplemental figure 10: Gene ontology analysis of CD26- NFs and CD26+ NFs reveal activation of similar pathways but with different players. A) Gene ontology analysis of the top-25 genes that define the cluster of CD26- NFs. B) Gene ontology analysis of the top-25 genes that define the cluster of CD26+ NFs. C) Genes expressed by CD26- and CD26+ NFs with the GO term 'Extracellular matrix'. Statistical significance was determined using Fisher's exact test with multiple comparison correction according to Benjamini-Hochberg.

Supplemental figure 11

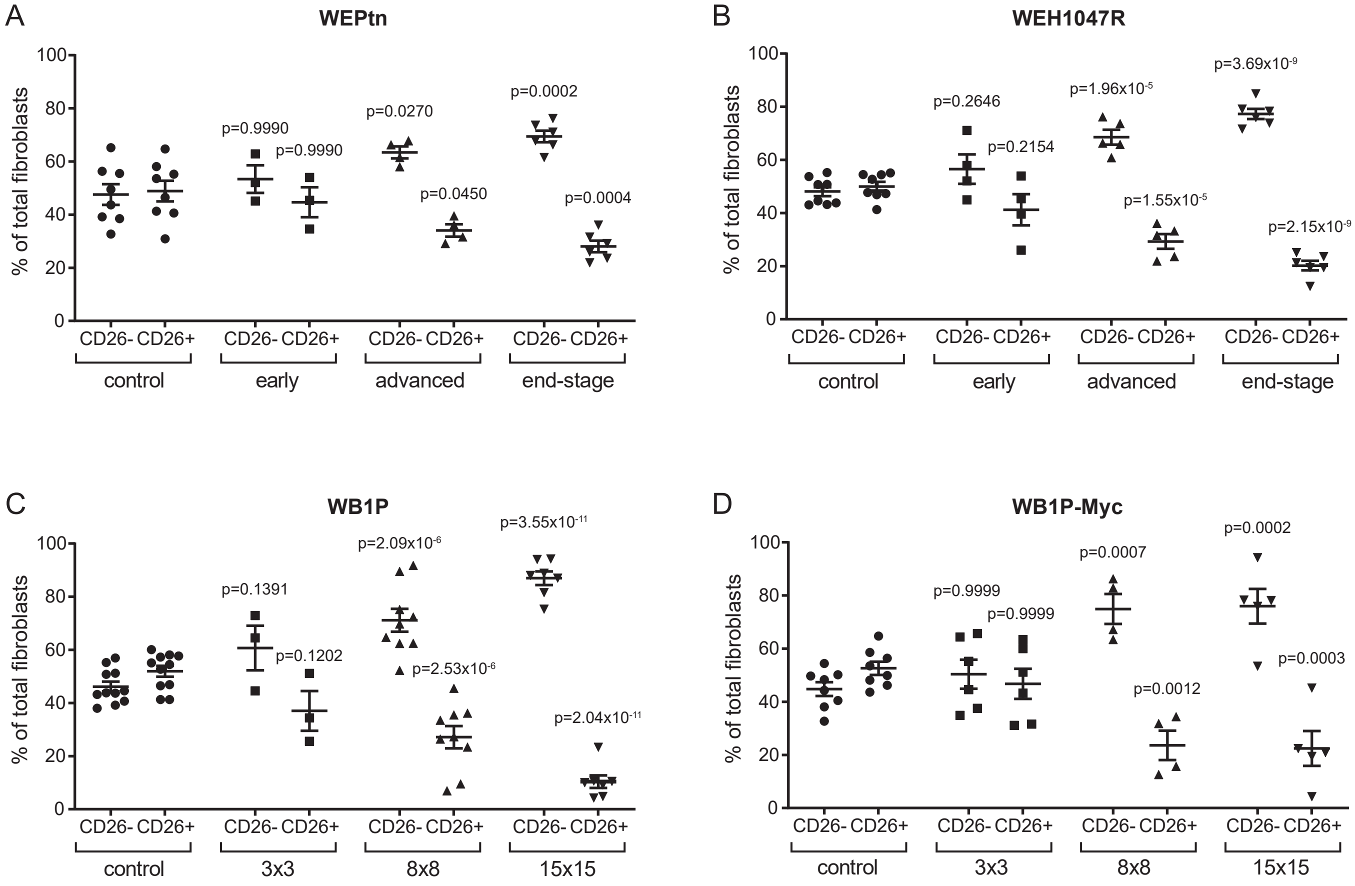

Supplemental figure 11: Balance between CD26- and CD26+ fibroblasts shifts as tumors progress. A) CD26- and CD26+ fibroblasts in WEPtn mice as determined by flow cytometry. ILC tumors were harvested at early (n=3), advanced (n=4) or end-stage (n=6) of tumor development. Together with mammary glands from control littermates (n=8) they were analyzed for CD26 expression. Fibroblasts were defined as EpCAM-/CD49f-/CD31-/CD45- cells. Within this population we determine the ratio of CD26- and CD26+ fibroblasts. B) CD26- and CD26+ fibroblasts in WEH1047R mice as determined by flow cytometry. Control mammary glands (n=8) and ILC tumors were harvested at early (n=4), advanced (n=5) or end-stage (n=6) of tumor development. C) CD26- and CD26+ fibroblasts in WB1P mice. Control mammary glands (n=11) and TNBC tumors were harvested at 3x3 mm (n=3), 8x8 mm (n=9) or 15x15 mm (n=7). D) CD26- and CD26+ fibroblasts in WB1P-Myc mice. Control mammary glands (n=8) and TNBC tumors were harvested at 3x3 mm (n=6), 8x8 mm (n=4) or 15x15 mm (n=5). For all panels the data is represented as mean ± SEM. A two-way ANOVA with Bonferonni multiple comparison correction was used to determine statistical significance by comparing CD26- or CD26+ NFs to the CD26- or CD26+ CAFs of the different time points, respectively.

Supplemental figure 12

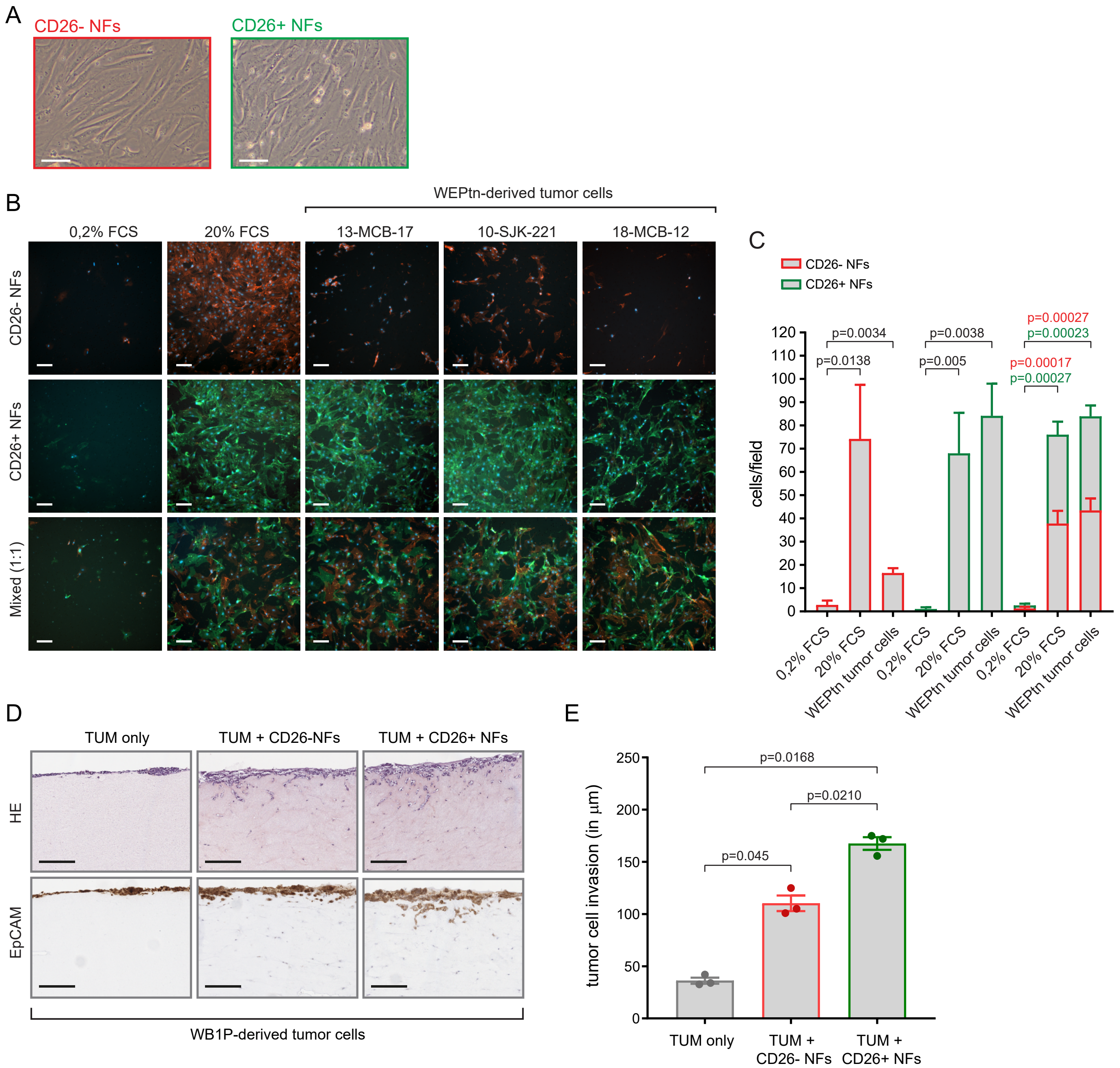

Supplemental figure 12: Recruitment of CD26- NFs towards WEPtn-derived ILC tumor cells depends on CD26+ NFs. A) bright field image of FACS-sorted CD26- and CD26+ mammary NFs. Image taken one week after sorting. Scale bars are 50 um. B) Transwell assay to assess the recruitment of CD26- NFs, CD26+ NFs or mixed CD26- and CD26+ NFs towards ILC tumor cells. Fibroblasts were harvested from mTmG mice by FACS and cultured for three days before switching the CD26+ NFs with Cre-encoding adenovirus to replace tdTomato expression with GFP. Four days post-switching the cells were plated in the recruitment assay. Representative result of five independent experiments with similar results. Scale bars are 150 um. C) Quantification of transwell assays in panel B (controls n=5, conditions with WEPtn tumor cells n=16). Data represented as mean ± SEM. Statistical significance was determined using student's t-test. D) Representative image of organotypic invasion assays performed with WB1P-derived tumor cells. Scale bars are 200 um. E) Quantification of organotypic invasion assays based on EpCAM staining (n=3 independent experiments with similar outcome). Invasion was measured in um from top of the gel to the invasive front of the tumor cells. Data represented as mean ± SEM. Statistical significance was determine using student's t-test.

Supplemental figure 13

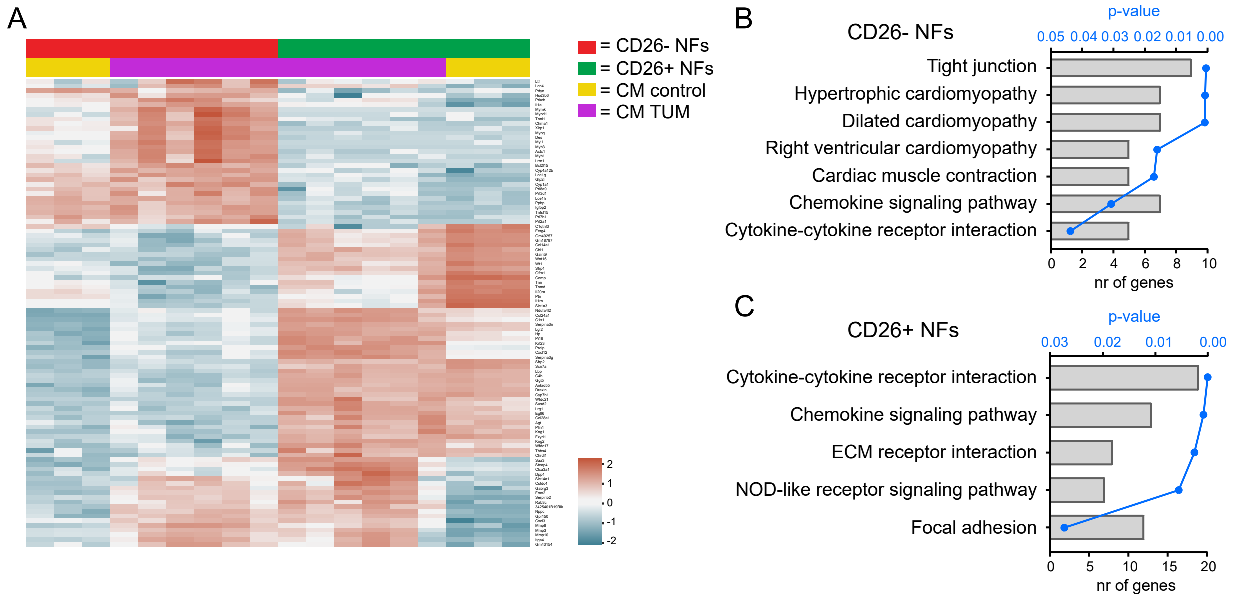

Supplemental figure 13: Differential response of CD26- and CD26+ NFs to tumor CM. A) Transcriptomics analysis and hierarchical clustering of CD26- and CD26+ NFs cultured in control CM (n=3) or WEPtn-derived tumor cell CM (n=6 different WEPtn cell lines). Top 100 most variable genes are displayed here. B) KEGG pathway analysis of significantly upregulated (Log2FC>2 and FDR<0.05) genes between CD26- NFs cultured in control CM vs CD26- NFs cultured in tumor CM. C) KEGG pathway analysis of significantly upregulated (Log2FC>2 and FDR<0.05) genes between CD26+ NFs cultured in control CM vs CD26+ NFs cultured in tumor CM. Statistical testing for panel B and C was done using Fisher's exact test with multiple comparison correction according to Benjamini-Hochberg.

Supplemental figure 14

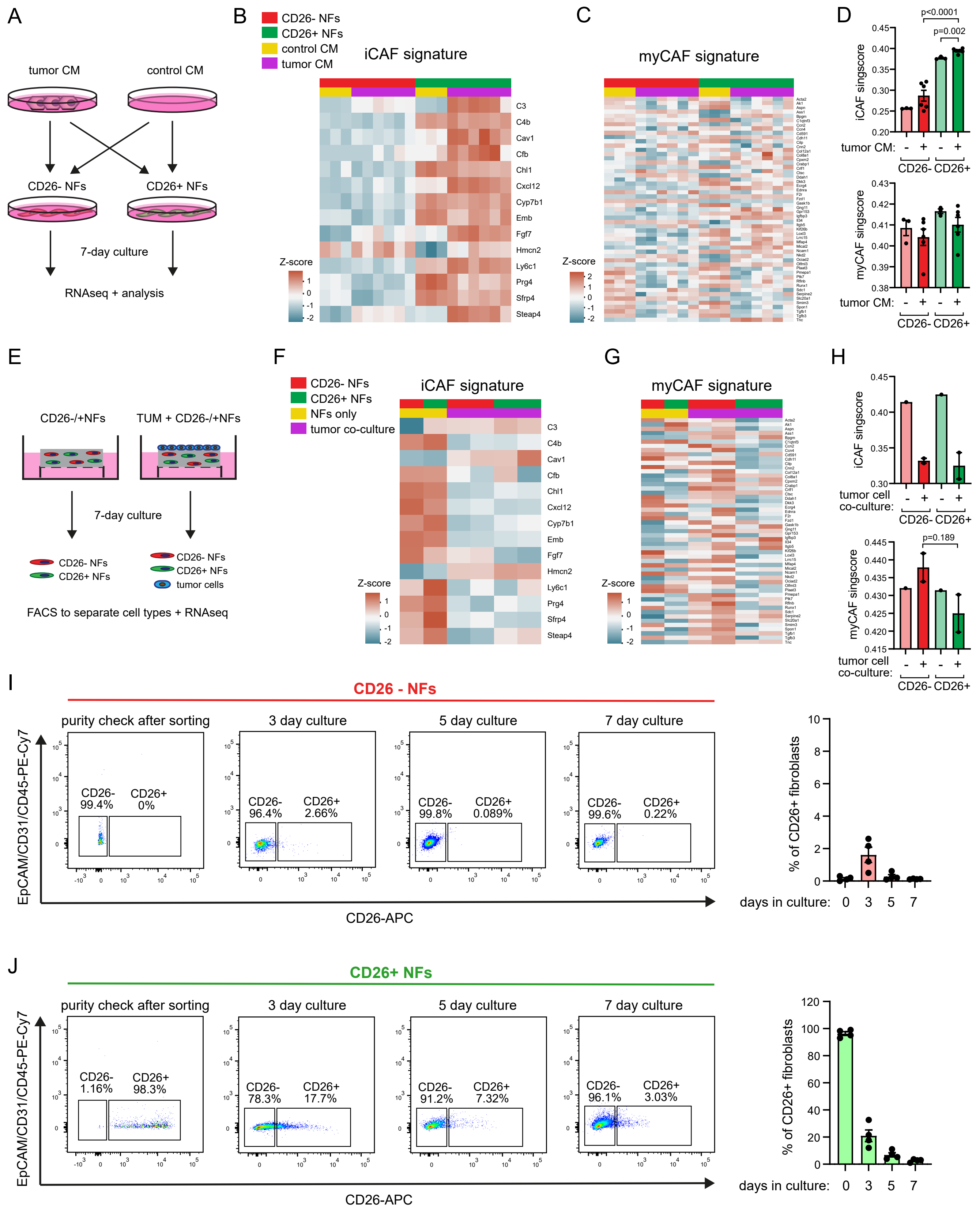

Supplemental figure 14: Cultured fibroblasts require differential culture conditions to induce and iCAF and myCAF phenotypes. A) Schematic overview of experimental set-up in which CD26- NFs and CD26+ NFs are cultured for 7 days in conditioned medium (CM) derived from 6 independent WEPtn-derived ILC tumor cell lines followed by RNA sequencing. B) Transcriptomics analysis of the iCAF gene signature in CD26- NFs and CD26+ NFs cultured in control CM or tumor CM. C) Analysis of the myCAF gene signature in CD26- NFs and CD26+ NFs cultured in control CM or tumor CM. D) Single sample gene set enrichment analysis of iCAF and myCAF signatures. Statistical significance was determined by two-tailed student's t-test. E) Schematic overview of experimental set-up in which CD26- and CD26+ NFs were cultured in collagen-containing matrix alone or in the presence of tumor cells (2 WEPtn-derived tumor cell lines) for 7 days. After 7 days the cell types were separated by FACS and sequenced. F and G) Analysis of the iCAF (panel F) and myCAF (panel G) gene signatures in CD26- and CD26+ NFs cultured alone or in presence of tumor cells. H) Single sample gene set enrichment analysis of iCAF and myCAF signatures. Statistical significance was determined by two-tailed student's t-test comparing CD26+ NFs and CD26- NFs in co-culture conditions with tumor cells. I-J) Flow cytometry analysis for CD26 expression of CD26- (panel I) and CD26+ (panel J) NFs directly after sorting (purity check after sorting) or after 3, 5 or 7 days in culture. Representative plots are shown together with graph showing results from 4 independent experiments with similar outcome. Data represented as mean ± SEM.

Supplemental figure 15

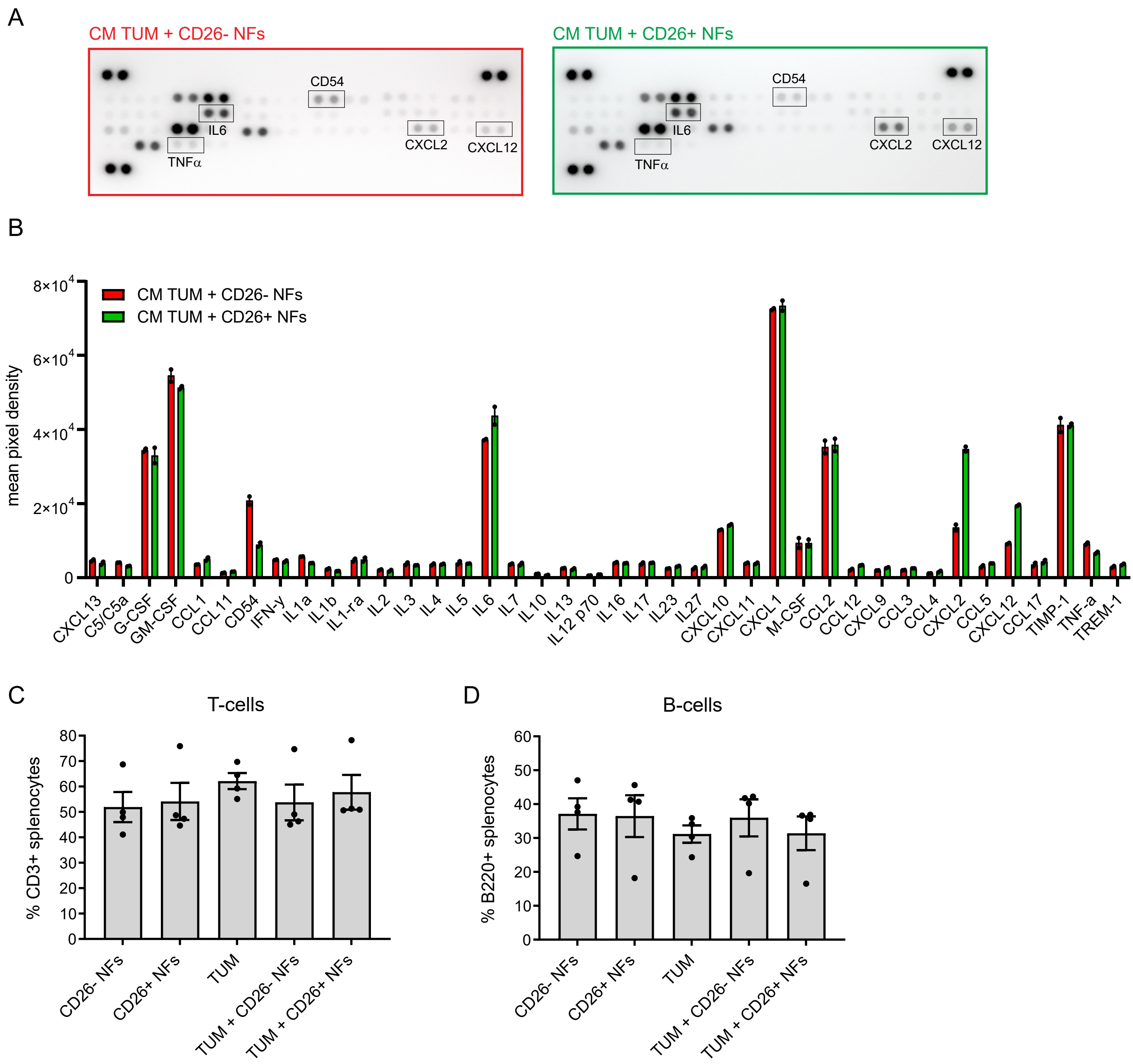

Supplemental figure 15: Differentially expressed cytokines between CD26- and CD26+ NFs do not impact recruitment of T- or B-cells. A) Results of cytokine array incubated with CM derived from three-day-co-cultures of CD26- NFs with tumor cells and CD26+ NFs with tumor cells. Differentially expressed proteins are boxed. B) Quantification of entire cytokine array as shown in panel A. Data represented as mean ± SEM. C and D) Results of transwell assays used to investigate recruitment of splenocytes towards the CM of fibroblast and tumor cell mono- or co-cultures. Migrated splenocytes were harvested from the bottom compartment after 24 hours, stained for CD3 and B220 to stain T- and B-cells, respectively and quantified by flow cytometry. Panel C shows migrated T-cells and panel D shows migrated B-cells. Results shown in panel C and D are from 4 independent experiments with similar outcome. Data represented as mean ± SEM.
