# Supplementary material for: CD26-negative and CD26-positive tissue-resident fibroblasts contribute to functionally distinct CAF subpopulations in breast cancer
Source: Nat Commun. 2023 Jan 12;14:183. doi: 10.1038/s41467-023-35793-w (PMC9837080; doi:10.1038/s41467-023-35793-w)
Supplement: Supplementary file 3 — Description of Additional Supplementary Files [file 41467_2023_35793_MOESM3_ESM.pdf]

Description of Additional Supplementary Files

File Name: Supplementary Data 1
Description: data file containing the following analyses and items:

- one-versus-rest cluster comparisons of data represented in Figure 3A.
- iCAF and myCAF gene signatures
- full Gene Ontology analysis of iCAFs, myCAFs, CD26- NFs and CD26+ NFs (related to main figure 3D-E and supplemental figure 10A-B)
- qPCR primer sequences for iCAF and myCAF markers (related to figure 3H and supplemental figure 7D).
