# Supplementary material for: CD26-negative and CD26-positive tissue-resident fibroblasts contribute to functionally distinct CAF subpopulations in breast cancer
Source: Nat Commun. 2023 Jan 12;14:183. doi: 10.1038/s41467-023-35793-w (PMC9837080; doi:10.1038/s41467-023-35793-w)
Supplement: Supplementary file 5 — Reporting Summary [file 41467_2023_35793_MOESM5_ESM.pdf]

nature portfolio

Corresponding author(s): Prof. Jos Jonkers

Last updated by author(s): Dec 7, 2022

Reporting Summary

Nature Portfolio wishes to improve the reproducibility of the work that we publish. This form provides structure for consistency and transparency in reporting. For further information on Nature Portfolio policies, see our Editorial Policies and the Editorial Policy Checklist.

Statistics

For all statistical analyses, confirm that the following items are present in the figure legend, table legend, main text, or Methods section.

n/a	Confirmed	
☐	☒	The exact sample size (*n*) for each experimental group/condition, given as a discrete number and unit of measurement
☐	☒	A statement on whether measurements were taken from distinct samples or whether the same sample was measured repeatedly
☐	☒	The statistical test(s) used AND whether they are one- or two-sided<br>*Only common tests should be described solely by name; describe more complex techniques in the Methods section.*
☒	☐	A description of all covariates tested
☐	☒	A description of any assumptions or corrections, such as tests of normality and adjustment for multiple comparisons
☐	☒	A full description of the statistical parameters including central tendency (e.g. means) or other basic estimates (e.g. regression coefficient) AND variation (e.g. standard deviation) or associated estimates of uncertainty (e.g. confidence intervals)
☐	☒	For null hypothesis testing, the test statistic (e.g. *F*, *t*, *r*) with confidence intervals, effect sizes, degrees of freedom and *P* value noted<br>*Give P values as exact values whenever suitable.*
☒	☐	For Bayesian analysis, information on the choice of priors and Markov chain Monte Carlo settings
☒	☐	For hierarchical and complex designs, identification of the appropriate level for tests and full reporting of outcomes
☐	☒	Estimates of effect sizes (e.g. Cohen's *d*, Pearson's *r*), indicating how they were calculated

Our web collection on statistics for biologists contains articles on many of the points above.

Software and code

Policy information about availability of computer code

Data collection	All data that required software-based analysis was generated in-house (transcriptomics, etc). No novel code or software was produced to analyze this data.
Data analysis	The following programs and versions were used thoughout this study: Matplotlib 3.3.4. Numpy 1.19.2. Pandas 1.2.3. Scanpy 1.7.1. Sinfo 0.3.1. IPython 7.21.0. Jupyter_client 6.1.7. Jupyter_core 4.7.1. Notebook 6.2.0. Python 3.7.10 (default, Feb 26 2021, 10:16:00) [Clang 10.0.0]. Darwin-19.6.0-x86_64-i386-64bit 8 logical CPU cores, i386. Session information updated at 2021-03-09 14:06. FlowJo v10.8.0. Qupath 0.3.0. ImageJ 1.53t. Imaris software version 9.8.

For manuscripts utilizing custom algorithms or software that are central to the research but not yet described in published literature, software must be made available to editors and reviewers. We strongly encourage code deposition in a community repository (e.g. GitHub). See the Nature Portfolio guidelines for submitting code & software for further information.

Data

Policy information about availability of data

All manuscripts must include a data availability statement. This statement should provide the following information, where applicable:

- Accession codes, unique identifiers, or web links for publicly available datasets
- A description of any restrictions on data availability
- For clinical datasets or third party data, please ensure that the statement adheres to our policy

The raw sequence data generated in this study is available at the Gene Expression Omnibus database under the following accession numbers: GSE205263 (https://

www.ncbi.nlm.nih.gov/geo/query/acc.cgi?acc=GSE205263) and GSE214933 (https://www.ncbi.nlm.nih.gov/geo/query/acc.cgi?acc=GSE214933). The publicly available human single-cell transcriptomics dataset used in this study is available in the Gene Expression Omnibus database under accession code GSE161529 (https://www.ncbi.nlm.nih.gov/geo/query/acc.cgi?acc=GSE161529). The publicly available laser-microdissected human ILC and IDC data used in this study is available in the Gene Expression Omnibus database under accession codes GSE148398 (ILC, https://www.ncbi.nlm.nih.gov/geo/query/acc.cgi?acc=GSE148398) and GSE68744 (IDC, https://www.ncbi.nlm.nih.gov/geo/query/acc.cgi?acc=GSE68744). The publicly available single-cell transcriptomics dataset of murine PDAC used in this study is available in the Gene Expression Omnibus database under accession code GSE129455 (https://www.ncbi.nlm.nih.gov/geo/query/acc.cgi). The remaining data are available in Supplementary data 1 and in the source data file.

Human research participants

Policy information about studies involving human research participants and Sex and Gender in Research.

Reporting on sex and gender	Any human data displayed in the manuscript (laser-microdissected tumor material from ILC and IDC patients, figure 4K-Q and supplemental figure 8G-J) has been published before (Gomez-Cuadrado et al., Cancers 2022 and Pal et al., EMBO J 2021). Information regarding sex, gender, recruitment, ethics and population characteristics is reported in the original publications.
Population characteristics	See above
Recruitment	See above
Ethics oversight	See above

Note that full information on the approval of the study protocol must also be provided in the manuscript.

Field-specific reporting

Please select the one below that is the best fit for your research. If you are not sure, read the appropriate sections before making your selection.

[x] Life sciences [ ] Behavioural & social sciences [ ] Ecological, evolutionary & environmental sciences

For a reference copy of the document with all sections, see nature.com/documents/nr-reporting-summary-flat.pdf

Life sciences study design

All studies must disclose on these points even when the disclosure is negative.

Sample size	Sample size of animal experiments were determined using power calculations based on pilot experimental data. In these power calculations we assumed normal distribution and used a power of 0.90 with an alpha of 0.05.
Data exclusions	The potential presence of significant outliers was determined using the Grubb's outlier test (Graphpad outlier calculator: https://www.graphpad.com/quickcalcs/Grubbs1.cfm alpha: 0.05) . No significant outliers were identified in the data presented in this study.
Replication	All experiments within this study were performed for a minimum of three independent experiments. All in vivo transplantation experiments contained at least 5 animals per group/timepoint. The single cell transcriptomic experiments were performed with at least 2 animals per time point/group.
Randomization	Randomization was applied where possible. Within the animal experiments limited randomization was applied as all animals needed to be of a certain genotype, age and sex. Mice transplanted with similar donor tissue were randomly allocated to the time point groups.
Blinding	Our laboratory animal technicians were blinded to the treatments/groups of mice while assessing tumor volumetric measurements. Measurements of invasion within the organotypic invasions assays and cell counts of the transwell assays were performed blinded by two researchers and results were compared and concordant. Flow cytometry analysis of tumors from transplantation studies were not blinded since information regarding the tumor model was needed to ensure the correct FACS panel of antibodies was added to the samples. monocyte recruitment assays were done blinded (re-numbering of wells) and analyzed by FACS blinded. All remaining experiment were not done blinded as information regarding samples was essential to conduct the experiments in a proper way.

Reporting for specific materials, systems and methods

We require information from authors about some types of materials, experimental systems and methods used in many studies. Here, indicate whether each material, system or method listed is relevant to your study. If you are not sure if a list item applies to your research, read the appropriate section before selecting a response.

Materials & experimental systems

n/a	Involved in the study
☐	☒ Antibodies
☐	☒ Eukaryotic cell lines
☒	☐ Palaeontology and archaeology
☐	☒ Animals and other organisms
☒	☐ Clinical data
☒	☐ Dual use research of concern

Methods

n/a	Involved in the study
☒	☐ ChIP-seq
☐	☒ Flow cytometry
☒	☐ MRI-based neuroimaging

Antibodies

Antibodies used

The following antibodies were used throughout this study (company, clone, order number):
EpCAM-PE-Cy7 (Invitrogen, ebioscience clone G8.8, 25-5791-80) dilution: 1:100
E-cadherin-PE-Cy7 (Biolegend, clone DECMA-1, 147310) dilution: 1:100
CD49f-AF700 (R&D systems, clone GoH3, FAB13501N-100UG) dilution: 1:100
CD45-AF700 (Invitrogen, ebioscience clone 30-F11, 56-0451-82) dilution: 1:100
CD45- BUV805 (BD biosciences, clone 30-F11, 748370) dilution: 1:100
CD45-FITC (Invitrogen, ebioscience clone 30-F11, 11-0451-82) dilution: 1:100
CD31-BUV395 (BD biosciences, clone 390, 740239) dilution: 1:100
PDGFRb-APC (Invitrogen, ebioscience clone APB5, 17-1402-82) dilution: 1:100
CD26-APC (Biolegend, clone H194-112, 137807) dilution: 1:100
CD26-PE (Biolegend, clone H194-112, 137804) dilution: 1:100
CD3-BUV395 (BD biosciences, clone 500A2, 740221) dilution: 1:100
B220-PE-Cy7 (Invitrogen, ebioscience clone RA3-6B2, 25-0452-82) dilution: 1:100
CD11b-APC (Invitrogen, ebioscience clone M1/70, 17-0112-82) dilution: 1:100
CD45-PerCP (BD biosciences, clone 30-F11, 557235) dilution: 1:100
Sca1- APC-Cy7 (BD biosciences, clone D7, 560654) dilution: 1:100
CD90.1-FITC (Invitrogen, ebioscience clone HIS51, 11-0900-81) dilution: 1:100
Keratin-14 (Biolegend (formerly Covance), polyclonal, PRB-155P) dilution: 1:700
Alpha-smooth muscle actin (Sigma-Aldrich, clone 1A4, A5228) whole mount analysis: 1:600. IF: 1:400. Western blot: 1:1000
CD26 (Abcam, clone EPR18215, ab187048) whole mount analysis: 1:200. western blot analysis: 1:1000
rat-anti-Keratin 8 (DSHB, clone TROMA-1) dilution: 1:200
rabbit-anti-Keratin 14 (Abcam, clone EPR17350, ab181595) dilution 1:1000
rabbit-anti-FSP1 (Abcam, EPR14639(2), ab197896) dilution 1:2000
PDGFR-beta (Cell signaling, 28E1, #3169) dilution 1:50
Alpha-smooth muscle actin (Fisher scientific, polyclonal, RB-9010) dilution 1:200
vimentin (Cell signaling, D21H3, #5741) dilution 1:200
EpCAM (Abcam, E144, ab32392) dilution 1:200
E-cadherin (Cell signaling, 24E10, #3195) dilution 1:200
anti-CXCL12 (R&D systems, 79014, MAB310) neutralizing ab concentration: 100 ug/ml
anti-CXCL2 (Thermo Fisher, 40605, MA5-23737) neutralizing ab concentration: 50 ug/ml
rabbit anti-C3 (Abcam, clone EPR19394, ab200999) dilution 1:1000
rabbit anti-TNC (Abcam, clone EPR4219, ab108930) dilution 1:1000
rabbit anti-TGFb1 (Abcam, clone EPR18163, ab179695) dilution 1:1000
mouse anti-Actin (Sigma-Aldrich, clone AC-15, A5441) dilution 1:2000
EnVision+ HRP-conjugated anti-rabbit (ready to use) (Dako Agilent, polyclonal, K400311-2) undiluted (RTU)
rabbit-anti-mouse HRP (Dako, polyclonal, P0260) dilution 1:2000
goat-anti-rabbit HRP (Dako, polyclonal, P0448) dilution 1:2000
goat-anti-mouse IgG conjugated to Alexa Fluor 488 (ThermoFisher, polyclonal, A-11001) dilution 1:1000
goat-anti-rabbit IgG conjugated to Alexa Fluor 568 (ThermoFisher, polyclonal, A-11011) dilution: 1:1000
goat-anti-rat IgG conjugated to Alexa Fluor 647 (ThermoFisher, polyclonal, A-21247) dilution 1:1000
goat anti-rabbit IgG conjugated to Alexa-647 (Thermo Fisher, polyclonal, A21245) dilution: 1:400
goat anti-mouse IgGconjugated to Alexa-647 (Thermo Fisher, polyclonal, A21241) dilution 1:400
donkey anti-rabbit conjugated to Alexa-568 (Thermo Fisher, polyclonal, A10042) dilution: 1:400
Details regarding the application and incubation can be found in the material and methods section.

Validation

All antibodies have been validated for the species (mouse) by the supplier and this is stated on their websites. All antibodies used for immunohistochemistry have been validated and extensively tested by the Experimental Animal Pathology Facility of the Netherlands Cancer Institute on various tissues including positive and negative controls. All antibodies used for FACS have been validated for the application and were possible tested on positive and negative control cell lines. In addition we compared if the percentage positive cells found for each population by FACS matched the population size assessed by immunohistochemistry.

Eukaryotic cell lines

Policy information about cell lines and Sex and Gender in Research

Cell line source(s)	Tumor cell lines used in this study were isolated from end-stage WEPtn- or WB1P-derived tumors. Normal fibroblasts (CD26-/+ NFs) were isolated by FACS from WapCre-negative Cdh1F/F;PtenF/F or mTmG (both FVB/n background) female mice between 8 and 16 weeks of age. Primary fibroblasts were kept in culture for no longer than passage 6 for experiments. Experiments were performed with the lowest passage number possible.
Authentication	WEPtn and WB1P-derived tumor cell lines were tested for their EpCAM expression by flow cytometry to ensure pure tumor cell cultures without contamination from other cell types. Only cultures with >90% EpCAM cells were used for experiments. Furthermore, the tumor cell lines were genotyped to ensure deletion of target driver genes (loss of E-cadherin and Pten in WEPtn cells and loss of Brca1 and Trp53 in WB1P cells). Primary normal fibroblasts were analyzed for PDGFR-beta expression by flow cytometry and displayed spindle cell shaped morphology. No genotyping was performed on the fibroblasts.
Mycoplasma contamination	All cell lines are regularly tested for mycoplasma in our lab and all cell lines and primary fibroblast cultures were tested negative for mycoplasma.
Commonly misidentified lines (See ICLAC register)	No misidentified cell lines were used in this study.

Animals and other research organisms

Policy information about studies involving animals; ARRIVE guidelines recommended for reporting animal research, and Sex and Gender in Research

Laboratory animals	The following strains were used for this study: WapCre;Cdh1F/F;PtenF/F (WEPtn), WapCre;Cdh1F/F;Col1a1invCAG-Pik3caH1047R-IRES-Luc (WEH1047R), WapCre;Brca1F/F;P53F/F (WB1P) and WapCre;Brca1F/F;Trp53F/F;Col1a1invCAG-Myc-IRES-Luc (WB1P-Myc). All tumor models were on FVB/n background and generated in-house. mTmG reporter mice were backcrossed for seven generations to FVB/n background to accommodate transplantations with donor tissue from our FVB/n-based breast cancer mouse models. EN1-Cre mice were purchased from The Jackson Laboratory (JAX stock number:007916) and backcrossed with mTmG (FVB/n) mice for 2 generations to generate EN1-Cre;mTmG mice for in vivo tracing. All mice were housed on standard 12 hour day/night cycle in individually ventilated cages with ad libitum food. Room temperature was maintained at 21 degrees Celsius and humidity was 55%. The age of the experimental animals dependent on the experiments. In brief: for MMEC transplantations the donor mice were between 4 and 6 weeks old and the recipient mice were between 18-21 days old. For the bone marrow transplantations the recipients were 8 weeks old at time of irradiation. Bone marrow donors were age- and gender-matched. For the whole mammary gland transplantation the donors and recipients were 4 weeks old at time of transplantation. Details are in the material and methods section.
Wild animals	No wild animals were used in this study.
Reporting on sex	All experiments were performed with female mice, as this research focuses on breast cancer.
Field-collected samples	This study did not use field-collected samples.
Ethics oversight	All animal experiments were approved by the Dutch Animal Ethical Committee and conducted in compliance with the Netherlands Cancer Institute and Dutch Animal Welfare guidelines.

Note that full information on the approval of the study protocol must also be provided in the manuscript.

Flow Cytometry

Plots

Confirm that:

- [x] The axis labels state the marker and fluorochrome used (e.g. CD4-FITC).
- [x] The axis scales are clearly visible. Include numbers along axes only for bottom left plot of group (a 'group' is an analysis of identical markers).
- [x] All plots are contour plots with outliers or pseudocolor plots.
- [x] A numerical value for number of cells or percentage (with statistics) is provided.

Methodology

Sample preparation	All tumors and control mammary glands were placed in PBS on ice upon harvesting. Samples were chopped into small pieces using a scalpel and processed into a single cell suspension using a digestion mix containing 2 mg/ml collagenase + 4ug/ml DNase in DMEM/F12. Samples were incubated for 60 minutes at 37°C under continuous shaking. After incubation the collagenase was inactivated by addition of equal volume of DMEM + 5% FCS. Samples were filtered through 70 um cell strainers and spun at 300g for 5 minutes to pellet the cells. Cell pellets were resuspended in red blood cell lysis buffer (RBC lysis, 155 mM NH_4Cl, 10 mM $KHCO_3$ anad 0.1 mM EDTA in H_2O) and incubated on ice for 5 minutes. Next the samples were spun down, 300g for 5 minutes at 4°C. Cell pellets were resuspended in FACS buffer (1% BSA + 5 mM EDTA in PBS) and

stained with appropriate antibodies for 30 minutes on ice. Next the samples were washed with FACS buffer, spun down and resuspended in FACS buffer prior to analysis.

Instrument

Flow cytometry analysis was done on a LSRII SORP flow cytometer equipped with 5 lasers (355 nm UV-laser, output setting 20 mW, 405 nm Violet laser, output 40 mW; 488 nm Blue laser, 50 mW,;561 nm Yellow-green laser, 40 mW and a 640 nm Red laser, 40 mW) from Becton Dickinson (BD), San Jose, CA, USA. Sorting of relevant cell populations within this study were done using a BD FACS Aria Fusion equipped with 4 lasers (405 nm Violet laser, output 85 mW; 488 nm Blue laser, 50 mW; 561 nm Yellow-green laser, 50 mW and a 638 nm Red laser, 100 mW). Cells were sorted using a 100um nozzle at a pressure of 20 psi.

Software

Software used for set-up and sorting: FACS DiVa version 8.0.1 from BD. Software used for analysis of samples run on LSRII SORP: FlowJo v10 from LLC

Cell population abundance

Sorting of CD26- and CD26+ NFs from normal mammary glands resulted in an average harvest of 1,2x10^6 cells of each population when the third, fourth and fifth mammary glands of 4 mice (age between 8 and 16 weeks) were pooled. On average the percentage of fibroblasts in the pooled mammary gland samples was 13,2% of total live single cells (roughly 50 % CD26- NFs and 50% CD26+ NFs). All samples were checked for purity post-sorting and the average purity was 95,6% and never below 89%.

Gating strategy

Gating strategy for analysis of fibroblasts in transplantation studies: FSC-A/SSC-A plots were used to gate cells, next FSC-A/FSC-H plots were used to select singlets. Next all Dapi-negative cells were selected as live single cells (FSC-A/DAPI plot). For WEPtn and WEH1047R tumors the next step was to plot EpCAM against CD45 to gate mammary epithelial cells (EpCAM+) and immune cells (CD45+). Cells negative for EpCAM and CD45 were gated to the next plot where endothelial cells were gated out (CD31+). EpCAM-/CD45-/CD31- cell were considered fibroblasts. Fibroblasts were analyzed for expression of PDGFRb expression or CD26 expression. tdTomato expression was determined for all populations. Gates were set using appropriate controls (unstained samples, single stained samples and FMO controls). A comparable gating strategy was used for WB1P and WB1P-Myc tumors. Here immune cells (CD45+) and endothelial cells (CD31+) were gated first. Cells negative for CD45 and CD31 were analyzed for EpCAM, E-cadherin and CD49f expression. Cells expressing E-Cadherin/EpCAM and CD49f were defined as mammary epithelial cells. CD45-/CD31-/EpCAM/E-cadherin-/CD49f- cells were fibroblasts. Additionally fibroblasts were analyzed for PDGFRb expression or CD26 expression. Also here, appropriate controls were used (unstained samples, single stained samples and FMO controls). Gates for tdTomato positive and negative gates were set using mammary tissue from mTmG (tdTomato+) reporter mice and FVB/n (tdTomato-) wildtype mice. Analysis and sorting of CD26- and CD26 + NFs relied on a similar gating strategy were fibroblasts were defined as EpCAM-/CD49f-/CD31-/CD45- cells. Fibroblasts were analyzed for their CD26 expression and CD26- and CD26+ fibroblast gates were set using a FMO control (sample stained with all relevant antibodies except CD26). The gating strategies used in this manuscript are shown in the supplemental figures 1 and 2.

☒ Tick this box to confirm that a figure exemplifying the gating strategy is provided in the Supplementary Information.
